# TMEA: A Thermodynamically Motivated Framework for Functional Characterization of Biological Responses to System Acclimation

**DOI:** 10.3390/e22091030

**Published:** 2020-09-15

**Authors:** Kevin Schneider, Benedikt Venn, Timo Mühlhaus

**Affiliations:** Computational Systems Biology, University of Kaiserslautern, 67663 Kaiserslautern, Germany; schneike@rhrk.uni-kl.de (K.S.); venn@rhrk.uni-kl.de (B.V.)

**Keywords:** GSEA, gene set enrichment analysis, pathway analysis, surprisal analysis, information theory, thermodynamics, free energy, acclimation response, transcription levels

## Abstract

The objective of gene set enrichment analysis (GSEA) in modern biological studies is to identify functional profiles in huge sets of biomolecules generated by high-throughput measurements of genes, transcripts, metabolites, and proteins. GSEA is based on a two-stage process using classical statistical analysis to score the input data and subsequent testing for overrepresentation of the enrichment score within a given functional coherent set. However, enrichment scores computed by different methods are merely statistically motivated and often elusive to direct biological interpretation. Here, we propose a novel approach, called Thermodynamically Motivated Enrichment Analysis (TMEA), to account for the energy investment in biological relevant processes. Therefore, TMEA is based on surprisal analysis, which offers a thermodynamic-free energy-based representation of the biological steady state and of the biological change. The contribution of each biomolecule underlying the changes in free energy is used in a Monte Carlo resampling procedure resulting in a functional characterization directly coupled to the thermodynamic characterization of biological responses to system perturbations. To illustrate the utility of our method on real experimental data, we benchmark our approach on plant acclimation to high light and compare the performance of TMEA with the most frequently used method for GSEA.

## 1. Introduction

Within the frame of their genetic capacity, organisms are able to acclimate to changes in environmental conditions. Acclimation responses thereby represent a complex dynamic adjustment of the entire molecular cellular network. The ability to acclimate ensures the survival of all living organisms and is therefore fundamental for the understanding of biological systems. Due to their mainly sessile lifestyle, plant systems particularly have to face fluctuating environmental conditions, including biotic and abiotic stresses [1,2]. Detailed knowledge about how plants acclimate to a changing environment is crucial especially in times of global climate changes, as plants are of great importance for our quality of life as a key source of food, shelter, fiber, medicine, and fuel [3,4]. A comprehensive understanding of plant acclimation responses allows the development of strategies to stabilize or enhance yields in increasingly hostile environments. Acclimation dynamics occur on different time scales—from minutes to days—and act on all system levels involving the modification of gene expression, protein activity, and metabolite profiles.

To elucidate these dynamics and to describe the different phases of acclimation, multiple time course experiments recording changes on various system levels have been performed in the past [5,6,7,8,9,10,11,12,13]. However, the identification and functional characterization based on these measurements remains a non-trivial task. Typically, these experiments result in huge lists of different molecules such as transcripts, metabolites, and proteins modified over the time course of the acclimation process. Therefore, gene set enrichment analysis (GSEA) has become an important approach to interpret these resulting lists. The principle of GSEA is to identify sets of biological molecules that are significantly overrepresented in a functional coherent set in a known biological pathway, compared to a background set of measured entities. Usually, the grouping is derived from functional gene and pathway annotation databases such as MapMan [14], GO [15], KEGG [16], Reactome [3], Wikipathways [17], BioCyc [18], or others.

One of the most frequently used approaches to perform GSEA is a one-sided hypergeometric or Fisher’s exact test that detects overrepresented functional sets derived from an experiment [19,20,21,22,23,24,25]. Therefore, every measured molecule is assigned a *p*-value or label that indicates whether it showed a (significant) change during a time course and/or compared to a reference. A subsequent hypergeometric test identifies functional sets that are significantly overrepresented in the data [26]. Every term leads to an individual test, leading to the necessity for multiple testing corrections. The drawback of this method is that it relies on applying a *p*-value cutoff to define the boundary between included and excluded molecules. This arbitrary distinction leads to a discretization of the information that dramatically influences the outcome of a GSEA [27] and is particularly difficult in time-series analysis. This problem is addressed by several methods that can be categorized into Functional Class Scoring (FCS) and Single-Sample (SS) methods. While FCS calculates scores (*p*-values or ranks) for every entity within a given set, SS aims to score every gene set per sample according to its importance [28,29,30,31]. In addition, multiple methods have been proposed to integrate multiple annotation databases or address the problem of overlapping set annotations due to molecules playing a role in different pathways and processes [32]. In addition, network-based approaches are available; however, they are restricted to biological systems where a deeper understanding of the molecular interaction is already available [33,34,35]. The existence of different counting or ranking metrics, enrichment statistics, and several variants on significance estimation demonstrates the difficulty of finding a single, optimal statistic due to the complexity, heterogeneity, and multi-modal distribution within the data [36]. Currently, the definition of an enriched pathway is predominantly of statistical nature due to an a priori defined set of interest. From a biological perspective, that might not always be an ideal scenario, especially if the pathways of interest are not regulated by a majority but rather a few or even a single key enzyme.

In this paper, we propose to account for the energy investment driving the required process to understand acclimation responses at the systems level. For this objective, we developed a novel approach called Thermodynamically Motivated Enrichment Analysis (TMEA). Plant systems are maintained in individual states far from thermodynamic equilibrium and fuel all biogeochemical processes by the absorption of incoming sunlight. Entropy production is a general consequence of these processes and allows computing their free energy. The principle of minimum entropy production states that systems are driven to steady states that are characterized by a minimum value of entropy production rate given the prevailing constraints [37].

Motivated by information theory, surprisal analysis offers a very compact, thermodynamic-free, energy-based representation of the biological steady state and of the biological change, the so-called unbalanced processes [38]. Therefore, we use surprisal analysis to compute free energy changes throughout the course of the specific acclimation response. Surprisal analysis identifies both a baseline state of maximum entropy and constraints that prevent the system from reaching it [39,40]. Molecules contribute to these constraints, and the difference in their contributions makes it possible to characterize different states of the system as patterns that collectively cause deviations from the baseline state. Associated with the constraints are time-dependent state variables that reflect the importance of the constraints and therefore carry information of how energy is invested over time [41,42]. In TMEA, we use the intensive variable *G*, which quantifies the contribution of each molecule underlying the free energy change as the basis for a Monte Carlo resampling procedure resulting in a functional characterization directly coupled to the thermodynamic characterization of biological responses to system perturbations, which is not yet addressed by conventional methods.

Finally, we demonstrate the application of our methods to light acclimation in *Arabidopsis thaliana* and evaluate the knowledge that we can recover solely from transcriptional changes compared to the current literature knowledge.

## 2. Materials and Methods

### 2.1. Dataset

The transcriptomics data used in this study were obtained from (NCBI Gene Expression Omnibus, Accession GSE125950) a high light experiment conducted with *Arabidopsis thaliana* [43]. First, 14-day-old Col-0 seeds were treated with 450 µmol photons m^−2^ s^−1^ for 4 days under long-day conditions (18 h d^−1^). After 4 days of acclimation, the light was reduced to control conditions (80 µmol photons m^−2^ s^−1^) for another 4 days. Entire shoots were harvested at 11 time points (0 min, 1 min, 15 min, 3 h, 2 days, 4 days for acclimation and de-acclimation, where 4 days of acclimation equals 0 min of de-acclimation). Transcripts were measured from three biological replicates for every time point by RNA-Seq using an Illumina HiSeq 2500 system (Illumina, San Diego, CA, USA). Metabolomics data for the verification of selected transcripts were obtained from the Supplemental Table S2 of the same study [43]. Metabolites were sampled at 13 time points (0 min, 5 min, 15 min, 3 h, 1 day, 2 days, and 4 days for acclimation and de-acclimation, respectively) [43].

### 2.2. Surprisal Analysis

Surprisal analysis (SA) assumes that a system will decrease its free energy spontaneously unless constrained [38]. It provides a method to determine a small set of state variables *λ_α_*, which are dependent on time and determine the deviations of the observed process from a balance state of minimal free energy. For every constraint, a weight is assigned to each measured entity (e.g., transcript, metabolite, or protein), which describes the influence of this molecule to the constraint.

The surprisal of each individual observation $X_{i}\left(t\right)$ is defined as the deviation from the steady state $X_{i}^{0}$:

(1) $$I\left(x_{i}\right)=-ln\left[\frac{X_{i}\left(t\right)}{X_{i}^{0}}\right].$$

Then, SA fits the surprisal by a sum of terms:

(2) $$-\underset{\alpha =1}{\sum }G_{i\alpha }\lambda_{\alpha }\left(t\right),$$

where α is the index of the constraint, $G_{i\alpha }$ is the weight of the event $X_{i}$ in constraint $G_{\alpha }$, and $\lambda_{\alpha }\left(t\right)$ is the Lagrange multiplier for $G_{\alpha }$ that is being varied to find the best fit. This is practically achieved by singular value decomposition, simultaneously yielding a baseline state of minimum free energy for α=0 [39,40,44].

Free energy changes can be determined for each constraint as work available to the molecular system under investigation from the results of surprisal analysis; the total work done on the system is the sum of these terms [45,46]:

(3) $$F_{\alpha }\left(t\right)=-\lambda_{\alpha }\left(t\right)\underset{i}{\sum }X_{i}\left(t\right)\;G_{i\alpha },\;F_{total}\left(t\right)=-\sum_{\alpha =1}\left(\lambda_{\alpha }\left(t\right)\sum_{i}X_{i}\left(t\right)\;G_{i\alpha }\right).$$

With an increasing constraint index, the contribution to the deviations from the baseline state drastically decreases. SA was computed using our implementation provided within the TMEA package [47] written in F# based on LAPACK Version 3.8 [48].

### 2.3. Functional Annotation and Pathway Database

Functional annotations for each transcript were obtained from MapMan ontology. MapMan is a plant specific ontology that covers functional annotations and pathway information in great detail. Entities sharing functional properties are summarized as a functionally annotated set (FAS) Mapping files are available at [49] for a collection of all MapMan terms and [50] for Arabidopsis-specific annotations. Metabolite annotations for each transcript were obtained from the KEGG Compound Database [51]. Compound-involved enzymes were mapped to transcript identifiers (TAIR 10) by using KEGG Orthology for *Arabidopsis thaliana* [52].

### 2.4. Gene Set Enrichment Analysis Based on Hypergeometric Function

Several methods for the identification of enriched FAS are summarized under the concept of gene set enrichment analysis (GSEA). One of the most established and frequently applied methods is a one-sided hypergeometric test, which detects overrepresented FAS in all FASs derived from the experiment [24,25]. For enrichment analysis based on hypergeometric tests, all genes were tested for significant differential expression during the time course. Differentially expressed genes (DEGs) were obtained using DESeq2 [53] by a comparison of transcripts at each time point of the high light treatment with the initial time point. Transcripts are labeled as DEGs if their abundance fold change is >2 with a false discovery rate (FDR) ≤ 0.05. A subsequent hypergeometric test identifies the FASs with a minimal size of 5 that are significantly overrepresented in the data [26]. Since one test is performed for each annotation, a multiple testing correction is performed by controlling the FDR by the Benjamini–Hochberg method [25,54,55].

### 2.5. Further Statistical Analysis and Visualization

All computational analyses were conducted using the open source F# libraries FSharp.Stats [56] and BioFSharp [57]. Linear regression, Benjamini–Hochberg correction, and clustering were conducted using the FSharp.Stats version 0.2.1-beta. For ontology annotation and GSEA based on hypergeometric tests, we used BioFSharp version 2.0.0-beta4 [57]. Data visualization was performed using the FSharp.Plotly version 2.0.0 chart library built on plotly.js [58].

## 3. Results

### 3.1. A Thermodynamic-Free Energy-Based Framework for the Functional Description of Biological Systems Not in Equilibrium Named TMEA

We present Thermodynamically Motivated Enrichment Analysis (TMEA), which coupled with surprisal analysis (SA) provides an unbiased functional description for the thermodynamic constraints prevailing on a biological system. It is based on thermodynamic and information theoretic principles and reduces the complexity of a given dataset using Monte Carlo simulation to a level that is both easier to manage and interpret from a biological point of view. Our open source implementation of TMEA in the functional programming language F# is freely available at https://github.com/CSBiology/TMEA [47].

TMEA applies three distinct steps: (i) the computation of SA to identify the constraints and contributing weights; (ii) the annotation and grouping of entities in the dataset using a given biological function pathway annotation databases, and (iii) a Monte Carlo permutation test performed by resampling of the weight sums as a test statistic for all functional sets. Testing assesses if the weight sum of each category is observed due to chance given the distribution of weight contributions provided by SA. We designed step (iii) specifically for the functional analysis of constraints reported by SA and here provide both a mathematical formulation and rationale of the design decisions.

Let $E=\left\{w_{1},\;\ldots ,\;w_{s}\right\}$ denote a set of cardinality s, containing weighted contributions $w_{i}$ of entities to the constraint $G_{\alpha }$. Let $E^{+}=\left\{w^{+}\in E:\;w^{+}>0\right\}$ and $E^{-}=\{w^{-}\in E:\;w^{-}<0\}$ denote the directional subsets of E with either positive or negative sign of cardinalities $s^{+}/s^{-}$. For the observed directional sums of contribution weights in $E^{+}$/$E^{-}$:

(4) $$\hat{w^{+}}=\sum E^{+};\;\hat{w^{-}}=\sum E^{-},$$

we want to compute the *p*-values

(5) $$p^{+}=P\left(W^{+}\geq \hat{w^{+}}\right);\;p^{-}=P\left(W^{-}\leq \hat{w^{-}}\right),$$

which determine how likely it is to observe contribution weight sums at least as extreme as $\hat{w^{+}}$/$\hat{w^{-}}$ for $E^{+}$/$E^{-}$ given the distribution of the test statistic for directional contribution weight sums $W^{+}$ and $W^{-}$. However, we do not know the exact distributions of $W^{+}$/$W^{-}$, which may also not be normal depending on the dataset. Additionally, estimating $W^{+}$ and $W^{-}$ by full permutation testing also proves impractical due to the size of the datasets typically used in modern biology. Therefore, we employ a Monte Carlo resampling procedure, which consists of resampling b independent replicates

(6) $$E_{1}^{*+},\;\ldots ,\;E_{b}^{*+};\;E_{1}^{*-},\;\ldots ,\;E_{b}^{*-}$$

from $G_{\alpha }$ with cardinality $s^{+}$ and $s^{-}$ and aggregating the sum of these samples as:

(7) $$W_{1}^{+},\;\ldots ,\;W_{b}^{+};\;W_{1}^{-},\;\ldots ,\;W_{b}^{-},$$

where

(8) $$W_{i}^{+}=\sum E_{i}^{*+},\;W_{i}^{-}=\sum E_{i}^{*-};\;i\in \left\{1,\;\ldots ,\;b\right\},$$

and using an empirical estimator for $p^{+}/p^{-}$:

(9) $$p_{empirical}^{+}=\frac{1}{b}\overset{b}{\underset{i=1}{\sum }}1\left\{W_{i}^{+}\geq \hat{w^{+}}\right\}\;p_{empirical}^{-}=\frac{1}{b}\overset{b}{\underset{i=1}{\sum }}1\left\{W_{i}^{-}\leq \hat{w^{-}}\right\}$$

where 1 is the indicator function. Note that b should be high, as the minimal *p*-value that can be obtained is $\frac{1}{b}$ [59]. After subsequently correcting $p_{empirical}^{+}/p_{empirical}^{-}$ based on FDR using the Benjamini–Hochberg method [55], the corresponding annotations can be assumed to have a significant influence on the respective constraint based on a confidence threshold of e.g., 0.05. A visual representation of the algorithm is depicted in Figure 1.

TMEA yields two functional descriptors for each constraint $G_{\alpha }$: one for positively contributing entities, and one for inversely contributing entities. These descriptors report what kind of functional information is overrepresented in either part of the constraint. Coupled with the constraint potentials $\lambda_{\alpha }$ obtained by SA, TMEA results can be used to further characterize the thermodynamic state transitions that the biological system undergoes while responding to a perturbation.

### 3.2. Contribution Weight Sums as Test Statistic

Ranking entities in a biological dataset from a thermodynamic point of view leads to a different perspective than applying purely statistical methods based on some form of majority voting [38]. The latter tend to reliably report FAS that show an overall consistent change but often fail to detect the importance of single or a small group of entities corresponding to a potential key regulator of the pathway. When statistically analyzing constraints reported by SA, it is important to select a test statistic that reflects this property. We applied TMEA to our high light acclimation benchmark dataset and treated positive and inverse weights separately after pooling the dominant constrains. Here, the first three constraints (α=1, …, 3) were considered to contain sufficient information to depict the characteristics of the high light response by an elbow criterion based on “importance loss” (Figure A2) between the singular values obtained by the singular value decomposition (SVD) procedure. Together with the baseline state (the “zeroth” constraint for α=0), these patterns are sufficient to recover 98.6% of the original data (Figure A2).

**Figure 2:**
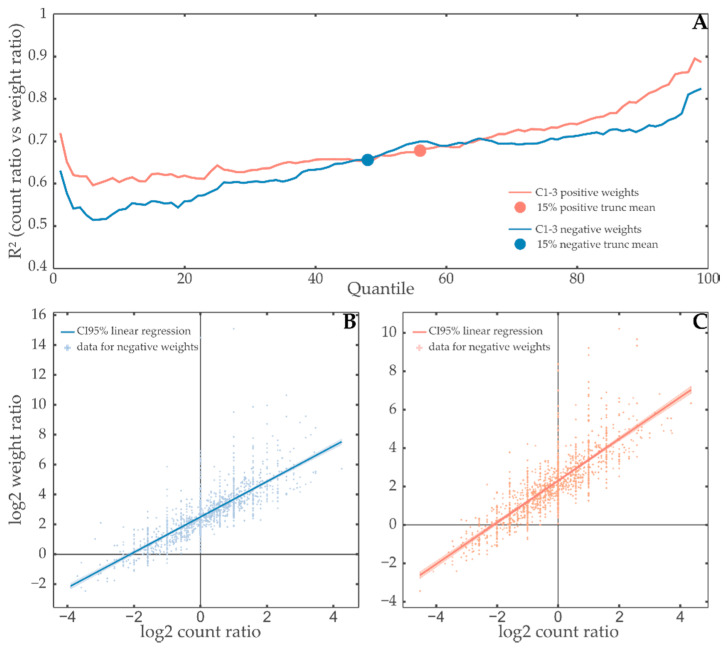
Contribution weights in constraints carry information beyond the count of extreme values. (**A**) R^2^ as a measure of linear regression quality of weight sum ratio (WR) by count ratio (CR) is shown in dependence of the quantile used to split the weight distributions of annotated sets to generate these ratios in either all positive (red) or all negative (blue) weight distributions for annotated subsets of constraints 1–3. The ±15% truncated mean of each is shown as a point of the same color. The quantile that separates weights in constraints 1–3 so that it produces the 15% truncated mean R^2^ regression quality shown in the upper part of the figure was used to split the weights in positively (56% quantile, (**C**)) and negatively (48% quantile, (**B**)) contributing parts of annotated subsets of constraints 1–3. Subsequently, both WR and CR were calculated for all the annotated subsets in the dataset. These values are shown as either red (right) or blue (left) points on the scatter plots. Linear regression was performed, and the resulting line was plotted with a 95% confidence band. These plots correspond to the regressions for a single y-value on the top plot. The existence and increase of outliers in the high weight/count ratio region suggests that high weight items carry an especially large amount of information that is lost when using traditional methods.

To quantify how counting extreme values might relate to the sum of weight contributions, we then calculated the weight threshold for all quantiles between 1% and 99%, and for all those thresholds, both the ratios of the sum of contribution weights (weight ratio (WR)) and the amount of weights above/below the threshold (count ratio (CR)) for all annotated sets (Figure 2 top). Subsequent investigation of the 15% trimmed mean of R^2^ of linear regression of WRs by CRs revealed that CRs can be used to explain 67.8% of the variance of WR for positively and 65.6% for negatively contributing subsets (Figure 2 bottom right and left, respectively), which indicates an importance of considering weights rather than just relying on counts. This observation supports the selection of the weight sum as the tests statistic for functionally describing constraints obtained by SA. Here, the directional sums of contribution weights $\hat{w^{+}}$/$\hat{w^{-}}$ can partially be explained with the count of extreme values suggesting that TMEA covers the classical scenario. However, a considerable amount of variance remains unexplained, pointing to the requirement to consider the influence of weights.

**Figure 3:**
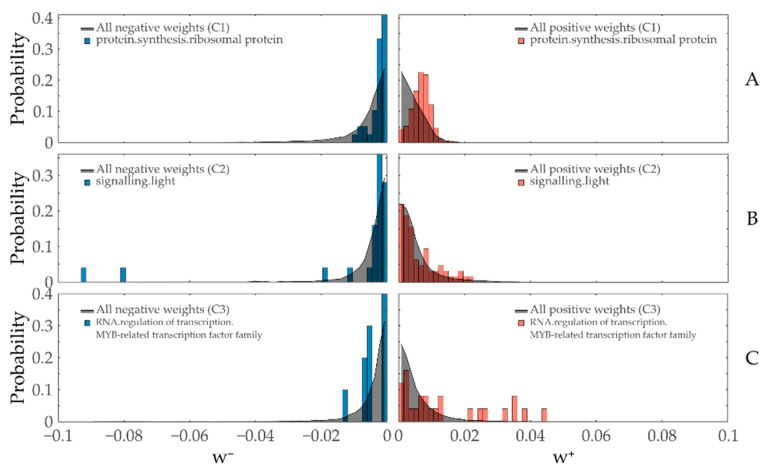
TMEA reports different weight distribution shapes for annotated subsets as significant. Histograms with a bin width of 0.0015 of both negatively (left part of the plots, blue) and positively (right part of the plots, red) contributing entities in the functionally annotated sets of (**A**) *protein.synthesis.ribosomal protein* for Constraint 1, (**B**) *signaling.light* for Constraint 2, and (**C**) *RNA.regulation of transcription.MYB-related transcription factor family protein* for Constraint 3 are plotted together with the respective overall distribution of weights (gray area plot) of the respective sign and constraint.

Based on these considerations, we can qualitatively classify three kinds of weight contributions: (1) cases where the overall distribution is shifted to more extreme values (i.e., the ‘majority vote’ case), (2) cases where a single or small amount of entities causes a whole functionally annotated set (FAS) to be reported as significantly altered, and (3) cases where a subset of the FAS is strongly skewed to extreme values, with cases (2) and (3) representing the aforementioned complementary results. Practical examples for each case are displayed in Figure 3. (1) The FAS *protein.synthesis.ribosomal protein* is reported to be significantly positively contributing to Constraint 1, with most of the entities being slightly more extreme than the overall weight distribution (Figure 3A), satisfying stoichiometric requirements during the regulation of large protein complexes [60]. Conversely, (2) *signaling.light* has a low amount of extreme contributions to Constraint 2, but two of them are sufficient to make the whole subset be reported as significant (Figure 3B). Finally, (3) the weights of a medium-sized subgroup of transcription factors in *RNA.regulation of transcription.MYB-related transcription factor family protein* show a distribution that is not reflected in the rest of the FAS (Figure 3C).

### 3.3. Comparison with Hypergeometric Test Based GSEA

**Figure A1:**
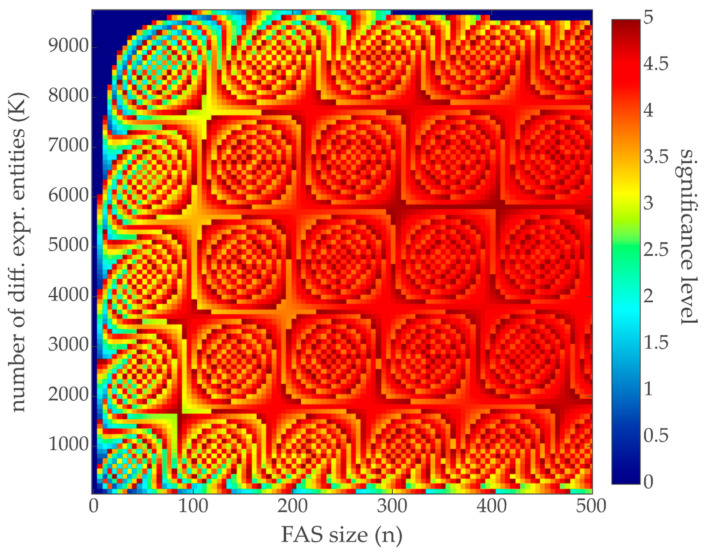
Maximal reachable α-level at a given α-level of 5%. The discrete nature of the hypergeometric distribution prevents the significance to reach 0.05 exactly. There always is a range of α-level space that must be sacrificed leading to a lower α than intended. The heatmap shows the maximal reachable α-level given: N = total number of genes = 10,000; K = number of differentially expressed genes; n = bin size; k = minimal number of differentially expressed genes needed for *p*-value < 0.05; intended α-level = 0.05. Especially when the bin size is low, even the half of the intended α-level often cannot be reached. Note that the bin size ranges from 1 to 500 in steps of 5.

In order to demonstrate the performance of the presented tandem approach, we compared our results from applying TMEA to transcriptomics data of a high light acclimation experiment to standard enrichment analysis based on hypergeometric distribution (hypGSEA). hypGSEA was performed for terms of transcripts that showed differential expression during the experiment time course (see Section 2.4). For TMEA, a statistical pre-analysis for binary entity labeling is not necessary, thereby eliminating bias resulting from preparatory analysis of the input. The size of entities grouped by one shared functional annotation often lies in the range of 5–50. Especially in small bin sizes (<50), the discrete nature of the hypergeometric distribution used in hypGSEA potentially leads to a lower significance level than intended (Figure A1). This loss of power could be mitigated by using a mid-*p*-value, which entails a risk of a significance level that is above the intended one [26,61] and therefore was not applied in this study.

**Figure 4:**
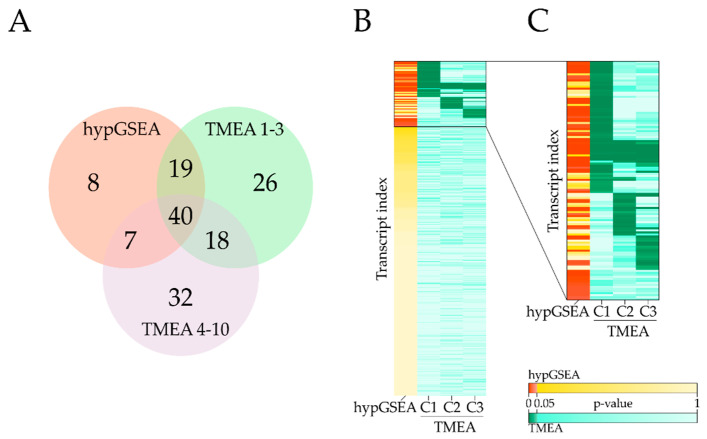
Comparison of significant functionally annotated sets (FASs) obtained by hypergeometric distribution (hypGSEA) and TMEA. (**A**) Venn diagrams of significant FASs with a minimum size of 5 reported by hypGSEA, TMEA constraints 1–3, and TMEA constraints 4–10 for a comparison without threshold. (**B**) Heatmap of adjusted *p*-values obtained by hypGSEA and TMEA. Measured transcripts were labeled with their respective hypGSEA *p*-value and the minimal TMEA *p*-value obtained within the first three constraints. All TMEA-significant bins are clustered by k-means clustering with k = 6. (**C**) Visualization of all FAS reported significant by hypGSEA and/or TMEA. Detailed cluster information is given in Table A1. Bins that are not reported by TMEA are appended to the end of the heatmap with increasing hypGSEA *p*-values.

On our light acclimation benchmark dataset, hypGSEA yields a set of 74 significant FASs. TMEA identified 103 FASs with significant contributions to constraints 1–3 and 97 FASs with a significant influence on constraints 4–10. Fifty-nine of the significant FASs are reported by both TMEA for constraints 1–3 and hypGSEA, leading to 15 FASs (12.7% of all reported FASs by hypGSEA and TMEA) exclusively reported by GSEA, and 44 exclusively reported by TMEA (37.3%) (Figure 4).

**Figure A2:**
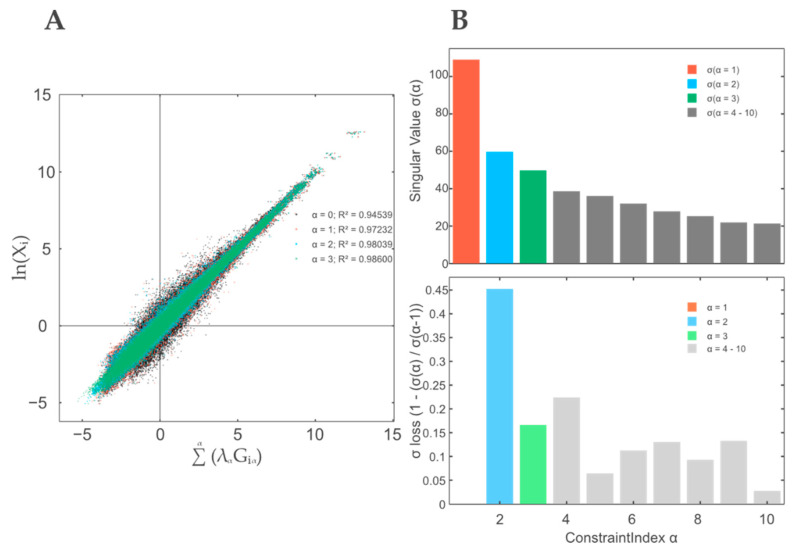
Constraint relevance. (**A**) Data reconstruction obtained by using (i) baseline state (constraint 0), (ii) Constraints 0–1, (iii) Constraints 0–2, and (iii) Constraints 0–3. (**B**) Singular values of constraints 1–10. The combination of a reconstruction efficiency of 98.6% and the singular value amplitude drop at α = 4 with no strong further decrease indicates a sufficient information supply by constraints 1–3.

Although the intersect of TMEA and hypGSEA significant FASs is large, no strong correlation between both *p*-values can be seen (Figure 4B,C). Especially, FASs that are reported to be significant in constraints with lower priority (constraints 2 or 3) show increased *p*-values for respective GSEA tests and vice versa. With an increasing constraint index, the relevance of FASs significantly contributing to the respective constraint diminishes. While the reported FASs show significant impact to these constraints, the constraints themselves may be of minor importance to the current condition. In a comparison without threshold, 39 unique FASs are reported by constraints 4–10 that are not contained in constraints 1–3 (Figure 4A). More than half (51.3%) of these FASs show a high functional similarity and differ only in the level of detail encoded by the depth within the ontology tree (Table S4). However, it is currently common practice to only consider constraints that account for the majority of information in the dataset (Figure A2) [38,41,44].

### 3.4. Case Study: Characterization of Light Acclimation in Arabidopsis thaliana

Since the understanding of a plant’s light response is of fundamental importance for future crop breeding and cultivation strategies, there has been a research focus on the acclimation to various light conditions, making light acclimation a suitable benchmark dataset. Furthermore, we focus on the transcripts as a proxy that influences the state of all levels: the proteome and, linked by proteins, the metabolome, lipidome, and even the phenome to some extent. So, most energy-consuming reactions or transitions are relying on transcripts, which makes them a feasible entry point to benchmark TMEA by relating observations previously not discovered on transcript but rather different system levels.

**Figure 5:**
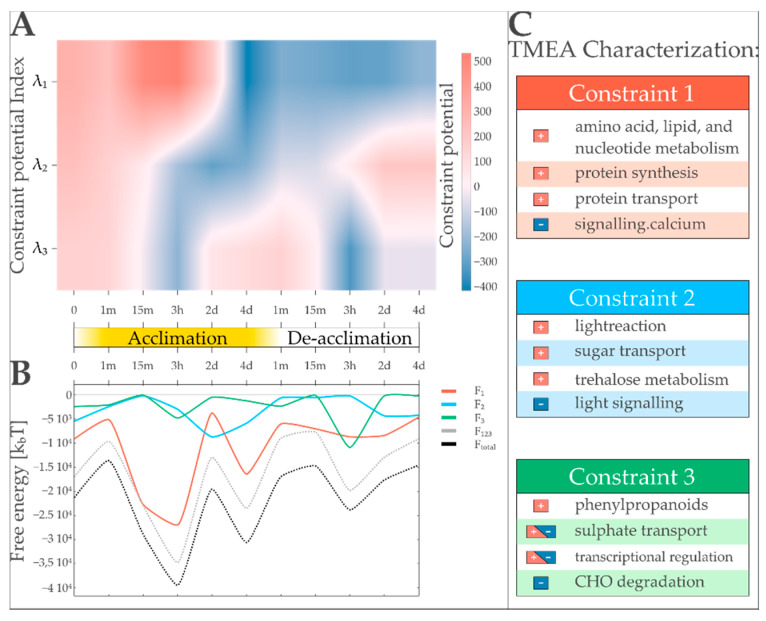
TMEA and surprisal analysis identify three major transcription patterns governing high light acclimation in *Arabidopsis thaliana* and provide a concise functional description for them. (**A**) Time course of the three major constraint potentials (λ_α_ for α = 1,2,3) indicate the importance of the respective transcription pattern. The potentials of the first three constraints (*λ*_1_–*λ*_3_) are shown for four days of acclimation and four days of subsequent de-acclimation. While *λ*_1_ separates the experiment in two major phases, *λ*_2_ and *λ*_3_ show more fluctuating patterns, defining three or four states, respectively. (**B**) Free energy landscapes defined by the three major state variables. Energy levels are plotted for transcription patterns (F_1_–F_3_), their sum (F_123_), and the total free energy when using all constraints for free energy calculation (F_total_). The dominant pattern is responsible for two of the three visible local energy minima. The least weighted pattern of the three is responsible for an energy minimum at the end of the time course. (**C**) Selected FASs reported by TMEA with significant influences on the respective constraints are listed. Directional influence (+ for positive, − for inverse) on the respective pattern is indicated.

TMEA analysis based on transcript amounts measured during light acclimation reveals functional descriptions for the different thermodynamic states of the biology identified by SA. The dominant state variable ($\lambda_{1}$) indicates the existence of two major states by undergoing a state transition (changing its sign) between two and four days of high light acclimation. This coincides with an energy investment governed by the first constraint (Figure 5B). Here, TMEA identifies major metabolic functions such as amino acid, lipid, and nucleotide metabolism as well as protein transport to be characteristic processes significantly contributing to energy investments. Calcium signaling shows the inverse contribution regarding the identified states of Constraint 1. In state variable $\lambda_{2}$, two state transitions seem to occur during the early phases of acclimation and de-acclimation, respectively (15 min to 3 h of treatment). A local energy minimum for this constraint can be observed at the same time as the state transition described by $\lambda_{1}$. The functional characterization of Constraint 2 by TMEA reveals a positive contribution of photosystem light reaction, sugar transport, and trehalose metabolism and an inverse contribution of light signaling. Three state transitions in $\lambda_{3}$ point to a more refined state shifting that subdivides the experimental time course into (1) an immediate acclimation response (0–15 min), (2) early acclimation (3 h), (3) late acclimation and condition change (2 days of acclimation to 15 min of de-acclimation), and (4) central de-acclimation (3 h to 4 days of de-acclimation). Naturally, the contributions of the third constraint to the overall free energy are low, but they are sufficient to be responsible for a third overall energy minimum at 3 h of de-acclimation. The dominant processes that characterize this constraint are major carbon degradation, sulfate transport, transcriptional regulation, and phenylpropanoid synthesis. In the following biological examination, we demonstrate that TMEA results obtained in our benchmark dataset seem to be biologically sound according to the current biological understanding of light acclimation.

#### 3.4.1. Anthocyanins

**Figure 6:**
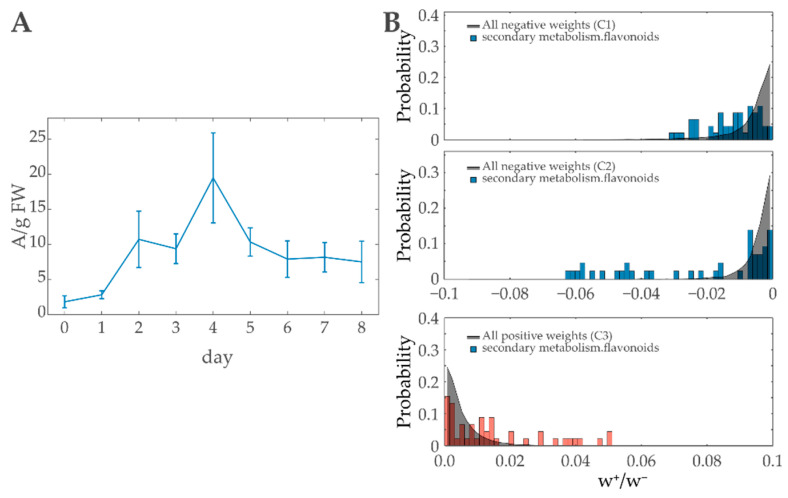
The role of Anthocyanins during high light treatment: (**A**) Anthocyanin content in *Arabidopsis thaliana* under 4 days of high light treatment (days 0–4) and 4 days of de-acclimation at ambient light condition (days 4–8). (**B**) Weight distributions of transcripts included in *secondary metabolism.flavonoids* demonstrating significant influences for constraints 1–3. TMEA reports a significance for the weight sums of all three constraints.

A well-known response to high light treatment in plants is the accumulation of anthocyanins, preventing photoinhibitory damage caused by high irradiance [62,63]. In photosynthetic active tissue, the dyes absorb excess radiation, thereby minimizing oxidative damage for e.g., the photosystems or DNA [63,64,65,66]. After onset of the highlight treatment, a significant anthocyanin accumulation was observed that increased during the 4 days of acclimation from ≈2 to $20\;A\cdot g\;FW^{-1}$ before decreasing to a constant level of ≈$8\;A\cdot g\;FW^{-1}$ during de-acclimation (Figure 6A).

The enrichment analysis in previous work [43] identified *flavonoid biosynthesis* to be significantly overrepresented in the same transcriptomics data utilized in this publication. Anthocyanins thereby are included due to the fact that flavonoids is a collective term for a huge variety of chemical compounds including anthocyanins [67]. hypGSEA using MapMan-Ontology also indicates an enrichment of the FAS *secondary metabolism.flavonoids, secondary metabolism.flavonoids.anthocyanins*, and further related FASs (Table S1). Light-protecting dyes have a significant role during high light response, ensuring the survival of the plant. TMEA recovers this importance by reporting anthocyanin and flavonoid-related FASs to be of significant importance in all considered major constraints (Figure 6B).

#### 3.4.2. Myb-Related Transcription Factor Family

A FAS solely detected by TMEA is *RNA.regulation of transcription.MYB-related transcription factor family*. Although based on the same dataset, neither the published enrichment [43] nor hypGSEA detected the respective FAS; however, biological relevance in high light response was discovered in previous studies. In [43], a motif search was performed within the 1000-bp promotor sequences of 456 genes and identified an overrepresented motif, which is bound by the members of Myb, and Myb-related-TF families, indicating a role in acclimation responses. The weights of the transcripts associated to this FAS were sufficient to report the importance in Constraint 3 using TMEA (see Figure 3C). The TF family is involved in the regulation of phenylpropanoid biosynthesis, which in turn is linked to lignin synthesis and UV protection [68,69]. Both hypGSEA and TMEA reported the phenylpropanoid biosynthesis to be enriched only taking transcripts into account. Particularly to Constraint 3, high weights are associated to both FASs (Table S2). As described in Section 3.4, the potential time course of Constraint 3 subdivides acclimation and de-acclimation in an early and late response (respectively).

**Figure 7:**
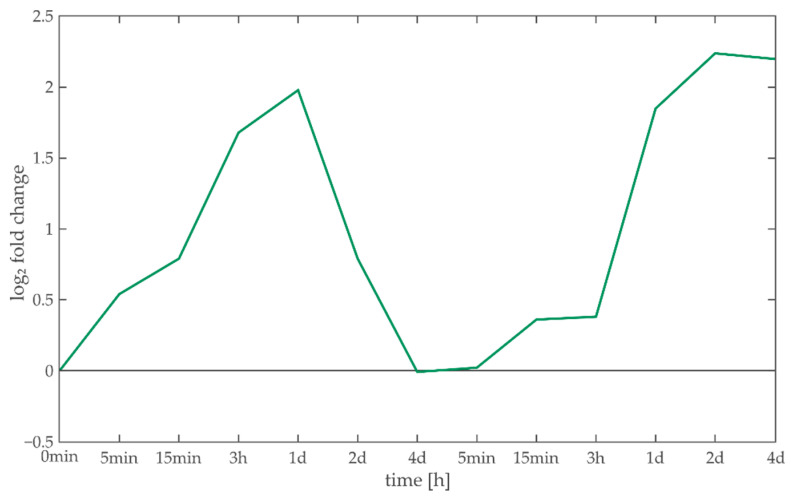
Phenylalanine time course. Phenylalanine fold changes during 4 days of high light acclimation and 4 days of de-acclimation under ambient conditions show increased abundance 3 h to 1 day after condition change.

One of the major metabolites that is required for phenylpropanoid synthesis and therefore is linked to Myb TF families is phenylalanine [69]. The metabolomics analysis conducted in parallel to the transcriptomics sampling reveals a distinct/prominent signal shape that quadrupled during the first day of acclimation, prior to returning to its original state during the high light phase. In the first day of the de-acclimation, the amount of phenylalanine quadrupled again and remained at high levels until the end of four days of de-acclimation. This characteristic shape resembles the time course of the potential of Constraint 3 (Figure 5A and Figure 7), where both phenylpropanoid biosynthesis and the Myb family show a significant importance. Of the 22 transcripts that can be assigned to phenylalanine metabolism by KEGG, 14 are directly associated to amino acid metabolism. Of the remaining eight transcripts, four can be assigned to phenylpropanoid synthesis.

#### 3.4.3. Ribosomes

Changes in environmental conditions make it necessary to rearrange the cellular proteome, which partially must be facilitated by the synthesis of new proteins at ribosomes. MapMan is exhaustive in the characterization and subdivision of ribosomal protein families. The measured transcripts are linked to 20 FASs related to *protein.synthesis.ribosomal protein*. Eight of these are associated to significantly enriched FASs in the TMEA analysis (Table S1) with nuclear as well as plastidic ribosome annotations among them. The third level FAS *protein.synthesis.ribosomal protein* contains 384 transcripts, of which 345 with positive weights to Constraint 1 show a characteristic shape (Figure 3A). Most of the weights show a constant shift toward higher influence, which is characteristic for protein complexes that rely on a stoichiometric relationship.

#### 3.4.4. Light/Calcium Signaling

Changes in the environment are perceived by plants and must be passed onto the responsible organs in order to take appropriate measures. Sometimes, it is sufficient to perform all steps within a single cell, so that the environmental information is perceived, processed, and reacted to without multi-cell communication [70]. Hormones and other signaling molecules serve as messengers for changes that must be communicated across several tissue types and functional units such as the shoot, root, or stem [71]. While the importance of three signaling-related FASs were identified by both hypGSEA and TMEA (*signlling*, *signaling.in sugar and nutrient physiology*, and *signaling.receptor kinases*), two additional FASs were reported exclusively by TMEA. Namely, *signaling.calcium* and *signaling.light* showed significant importance to constraint 2 or 3 respectively.

In FAS *signaling.light*, two genes were given particularly high weights. These two genes are *early light-induced protein 1* (ELIP1) and ELIP2 (AT3G22840 and AT4G14690), which both show a high upregulation upon high light treatment [72,73]. In fact, ELIP2 shows the overall highest negative weight in Constraint 2. Both are regulated by UVR8 [74] and CRY1 [73]. They are supposed to protect the plant cells from photo-oxidative stress [75,76] and play an important role in chlorophyll synthesis regulation [77].

Calcium ions are one of the most used intracellular second messengers in plants. Many environmental conditions trigger calcium-dependent signaling cascades, eventually leading to the activation of kinases responsible for appropriate stress responses [78,79]. TMEA identified the negative FAS weights to be significant in the most contributing constraint (constraint 1).

## 4. Discussion

Evaluating the performance of a GSEA method is challenging, as it is difficult to know which gene sets should be considered as true positives. A common approach is to simulate data to validate a particular method [80,81,82]. However, the validity of this approach is debatable, as the model used for the simulation strongly influences the results [28].

In this paper, we presented a novel approach to gene set enrichment analysis that is based on surprisal analysis (SA) and captures both biological functional knowledge and thermodynamic state description. We presented our rationale and formulation of the approach and applied it comparatively to hypergeometric test-based GSEA on a large transcriptomic dataset. To that extend, we could show that our proposed method can recover the functional knowledge extracted by the GSEA methods most frequently applied in comparable studies. Furthermore, we were able to report an array of additional biologically relevant findings based on transcriptional changes only that are in line with current literature knowledge and evidently emerge from its thermodynamic substantiation. For systemic acclimation responses, a proteome rearrangement is fundamental and well-studied. While under high light conditions, light harvesting is of minor importance, energy handling, energy distribution, and light protection become critical. Photoprotective mechanisms must be activated immediately without transcriptional reorganization and an extensive loss of time, so prearranged mechanisms are activated by post-translational modifications [83,84]. On the other hand, long-term and non-vital responses required within seconds can be regulated translationally. Most if not all reactions/transitions within an organism have their fundamental cause in the generation of catalyzing enzymes, whose abundances are in turn realized by transcriptional changes. It should be stressed though that this approach to validate TMEA is by no means perfect, as the process of previous knowledge discovery can also be biased by the methods applied by the different authors; however, it is thoroughly manually evaluated by an expert community.

Additionally, we believe that our approach is especially suited to analyze acclimation response on a systems level. Since biological systems always are under change, e.g., because of developmental issues or circadian rhythms, often a reference is desired to which the treated organism is compared. Two common procedures rely on (i) a control organism/culture monitored simultaneously to the treated one or (ii) a specific time point prior to the treatment that is taken as reference for the identification of condition responses. Both methods lack in robustness since (i) treated organisms behave in a different manner compared to control organisms, especially when treated with a systemic disturbance or during phases of development, and (ii) a single reference point can lead to massive misjudgments if the measurements are affected by an experimental bias. In previous studies, it could be shown that a thermodynamic viewpoint using SA alone already improves the understanding of responses to systems perturbation in plants [85,86,87]. However, we could demonstrate in this work that while SA is able to reveal states of the transcription system during acclimation, TMEA elucidates the subjacent pathways, contributing to these states. Thereby, TMEA provides a thermodynamic interpretation of the importance of functionally annotated sets (FASs).

In our transcript dataset, this leads to the novel finding of three stable states during light acclimation of *Arabidopsis thaliana* and allows for the distinction of functionally different phases during the acclimation response. The first stable state at 3 h of perturbation (Figure 5B) indicates an energy-intensive early acclimation phase, coinciding with the highest overall energy dissipation of the transcript system. To this state, only the first state variable is contributing meaningfully. TMEA characterization of the first transcription pattern informs that the energy sinks of the transcription system for this state are mainly metabolic pathways and protein synthesis, with a focus on ribosomal proteins (Figure 5 right, Table S1). The second stable state of the transcript system is identified at the last time point of acclimation treatment (4 days, Figure 5B) and can be interpreted as the acclimated state of the system, where energy is invested in the same pathways as in the first stable state, but possibly to maintain the long-term acclimation. The third stable state is reached in the early phase of de-acclimation (3 h, Figure 5B), with the third transcription pattern as the main energy sink. One of the central functions characterized to be significantly contributing to this transcription pattern is that of the various transcriptional regulators (Figure 5 right, Table S1). We hypothesize that this may be an indication for priming [88] of the transcript system for future responses to high light conditions. It is important to note that the energy investments in Transcription Pattern 2 are not leading to local energy minima. Interestingly, the time point at which the most work is done by this pattern (2 days into the acclimation phase of the experiment, Figure 5B) coincides with an overall local energy maximum, therefore lowering the overall energy level of the transcription system at this point. TMEA functionally associates this pattern mainly with light signaling and light reaction-related pathways (Figure 5 right, Table S1). These functional characterizations together with the fact that this pattern is not responsible for stable states leads us to the assumption that it is mainly responsible to lower the energy barriers that have to be overcome by the transcript system to reach its stable states, indicating that TMEA can separate regulatory patterns from enzymatic ones.

For future work, it might be beneficial to extent TMEA for the analysis of multivariate datasets using the multivariate version of the SA [89]. This would allow integrating information from different systems levels for the thermodynamically motivated functional characterization of biological responses to system acclimation. Furthermore, additional—and more practical—knowledge may be gained when comparing TMEA characterizations of different plants over the same condition, especially when applied to crop species or even organisms from another branch of life. So far, we provide an implementation of the whole analysis framework to facilitate the application of TMEA on different datasets using specific functional gene and pathway annotation databases. As more knowledge is collected and curated in those databases, we believe that TMEA will be increasingly useful for researchers especially studying systems acclimation responses.

## Figures and Tables

**Figure 1:**
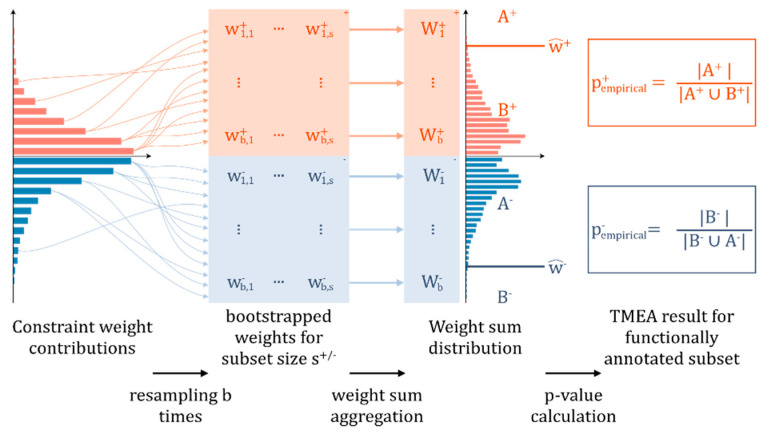
Schematic overview of the Monte Carlo permutation testing procedure used in Thermodynamically Motivated Enrichment Analysis (TMEA). Left to right: For a functionally annotated set of size s (s>5) in the original dataset, the size of the positively and negatively contributing subsets is determined ($s^{+/-}$). Subsequently, b random samples are resampled from the weight distribution of the original constraint yielded by surprisal analysis from either the positive or negative part respectively, to generate *b* bootstrapped samples of sizes $s^{+/-}$. Then, these samples are aggregated to generate b weight sums for positive and negative weights each. Then, the frequency distributions of these weight sums are used to report empirical *p*-values, which inform how likely it is to observe the given positive or negative weight sum for bin sizes $s^{+/-}$ in the original constraint by chance based on the values above ($A^{+/-}$) and below ($B^{+/-}$) the observed value.

## Supplementary Materials

The following are available online at https://www.mdpi.com/1099-4300/22/9/1030/s1, Table S1: Comparison of significantly contributing FAS, Table S2: Detailed TMEA result, Table S3: Detailed hypGSEA result, Table S4: Comparison of significantly contributing FASs in TMEA.

## Abbreviations

The following abbreviations are used in this manuscript:

DEG	Differentially expressed gene
FAS	Functionally annotated set
FCS	Functional Class Scoring
FDR	False discovery rate
GSEA	Gene set enrichment analysis
hypGSEA	Gene set enrichment analysis based on hypergeometric tests
TMEA	Thermodynamically motivated enrichment analysis
SA	Surprisal analysis
SS	Single-Sample
SVD	singular value decomposition

## Appendix A

**Table A1:** Significant FASs reported by TMEA in constraints 1–3. The *p*-values were clustered using the k-means clustering algorithm with a cluster number of 6 (cluster ID 1–6). The corresponding heatmap is depicted in Figure 4.

cID	MapMan Annotation (FAS)	cID	MapMan Annotation (FAS)
1	cell wall.cell wall proteins	4	major CHO metabolism
1	cell wall.cell wall proteins.AGPs	4	major CHO metabolism.degradation
1	cell wall.cell wall proteins.AGPs.AGP	4	major CHO metabolism.degradation.starch
1	cell wall.pectin*esterases.misc	4	misc.invertase/pectin methylesterase inhibitor family protein
1	lipid metabolism.FA desaturation	4	not assigned.no ontology.DC1 domain containing protein
1	lipid metabolism.FA desaturation.desaturase	4	not assigned.unknown
1	misc.beta 1,3 glucan hydrolases	4	RNA.regulation of transcription.AP2/EREBP, APETALA2/Ethylene-responsive element binding protein family
1	misc.beta 1,3 glucan hydrolases.glucan endo-1,3-beta-glucosidase	4	RNA.regulation of transcription.C2C2(Zn) CO-like, Constans-like zinc finger family
1	misc.glutathione S transferases	4	RNA.regulation of transcription.C2C2(Zn) DOF zinc finger family
1	misc.nitrilases, *nitrile lyases, berberine bridge enzymes, reticuline oxidases, troponine reductases	4	RNA.regulation of transcription.MYB-related transcription factor family
1	misc.O-methyl transferases	4	RNA.regulation of transcription.Psudo ARR transcription factor family
1	misc.protease inhibitor/seed storage/lipid transfer protein (LTP) family protein	4	secondary metabolism.isoprenoids.terpenoids
1	nucleotide metabolism.synthesis.purine	4	secondary metabolism.phenylpropanoids.lignin biosynthesis
1	protein	4	stress.abiotic
1	protein.degradation.AAA type	4	stress.abiotic.cold
1	protein.synthesis	4	stress.biotic.respiratory burst
1	protein.synthesis.ribosomal protein	4	transport.sulfate
1	protein.synthesis.ribosomal protein.eukaryotic	5	cell wall
1	protein.synthesis.ribosomal protein.eukaryotic.40S subunit	5	cell wall.modification
1	protein.synthesis.ribosomal protein.eukaryotic.60S subunit	5	misc
1	protein.synthesis.ribosomal protein.prokaryotic.chloroplast	5	secondary metabolism
1	protein.synthesis.ribosomal protein.prokaryotic.chloroplast.50S subunit	5	secondary metabolism.flavonoids
1	protein.synthesis.ribosome biogenesis	5	secondary metabolism.flavonoids.anthocyanins
1	protein.synthesis.ribosome biogenesis.Pre-rRNA processing and modifications	5	secondary metabolism.flavonoids.anthocyanins.anthocyanin 5-aromatic acyltransferase
1	protein.synthesis.ribosome biogenesis.Pre-rRNA processing and modifications.snoRNPs	5	secondary metabolism.flavonoids.dihydroflavonols
1	protein.synthesis.ribosome biogenesis.Pre-rRNA processing and modifications.WD-repeat proteins	5	stress
1	redox.glutaredoxins	5	stress.biotic
1	RNA.regulation of transcription.ARR	5	transport
1	RNA.regulation of transcription.NAC domain transcription factor family	6	cell wall.degradation
1	RNA.regulation of transcription.WRKY domain transcription factor family	6	cell wall.degradation.mannan-xylose-arabinose-fucose
1	secondary metabolism.simple phenols	6	DNA.synthesis/chromatin structure.retrotransposon/transposase
1	signaling	6	DNA.synthesis/chromatin structure.retrotransposon/transposase.gypsy-like retrotransposon
1	signaling.in sugar and nutrient physiology	6	hormone metabolism
1	signaling.receptor kinases.DUF 26	6	hormone metabolism.auxin
1	signaling.receptor kinases.misc	6	minor CHO metabolism
1	signaling.receptor kinases.wall associated kinase	6	minor CHO metabolism.trehalose
1	signaling.receptor kinases.wheat LRK10 like	6	minor CHO metabolism.trehalose.potential TPS/TPP
1	stress.biotic.PR-proteins.plant defensins	6	misc.gluco-, galacto- and mannosidases
1	transport.Major Intrinsic Proteins	6	not assigned.no ontology.glycine rich proteins
2	amino acid metabolism.synthesis	6	not assigned.no ontology.pentatricopeptide (PPR) repeat-containing protein
2	amino acid metabolism.synthesis.aspartate family	6	PS.lightreaction
2	development.storage proteins	6	PS.lightreaction.photosystem II
2	hormone metabolism.auxin.induced-regulated-responsive-activated	6	PS.lightreaction.photosystem II.LHC-II
2	nucleotide metabolism.synthesis	6	secondary metabolism.flavonoids.chalcones
2	protein.synthesis.ribosomal protein.eukaryotic.60S subunit.L7A	6	secondary metabolism.flavonoids.flavonols
2	protein.synthesis.ribosomal protein.prokaryotic	6	secondary metabolism.phenylpropanoids
2	signaling.calcium	6	signaling.light
2	stress.biotic.receptors	6	transport.ABC transporters and multidrug resistance systems
2	transport.Major Intrinsic Proteins.PIP	6	transport.sugars
3	misc.cytochrome P450		
3	misc.GDSL-motif lipase		
3	misc.peroxidases		
3	signaling.receptor kinases		
3	stress.biotic.PR-proteins		
